# Miscellany

**Published:** 1909-11

**Authors:** 


### MISCELLANY.

Medical Supervisor.—Indian service (Field). The United States Civil Service Commission announces an examination on November 24, 1909, at the usual places, to secure eligibles from which to make certification to fill a vacancy in the position of medical supervisor in the Indian field service at $250.00 a month and expenses, and vacancies requiring similar qualifications as they may occur in that service, unless it shall be decided in the interests of the service to fill the vacancy by promotion, reinstatement, or transfer.

The examination will consist of the subjects mentioned below, weighted as indicated: (1) Letter-writing (medical subject) 5; (2) Anatomy and physiology, 5; (3) Chemistry, materia medica, and therapeutics, 5; (4) Surgery, general and special, 10; (5) Hygiene and practice of medicine, 15; (6) Pathology and bacteriology, 10; (7) Training and experience, with special reference to tuberculosis and trachoma, 50; total, 100.

Candidates should at once apply either to the United States Civil Service Commission, Washington, D. C., or to the secretary of the board of examiners at the usual places, for application form 1312. No application will be accepted unless properly executed and filed with the Commission at Washington, prior to the hour of closing business on November 13, 1909. In applying for this examination the exact title as given at the head of this announcement should be used in the application.

Issued October 12, 1909.

